# Effect of Statin Use on Outcomes of Adults with Candidemia

**DOI:** 10.1371/journal.pone.0077317

**Published:** 2013-10-14

**Authors:** Guillermo Cuervo, Carolina Garcia-Vidal, Marcio Nucci, Francesc Puchades, Mario Fernández-Ruiz, Analía Mykietiuk, Adriana Manzur, Carlota Gudiol, Javier Pemán, Diego Viasus, Josefina Ayats, Jordi Carratalà

**Affiliations:** 1 Hospital Universitari de Bellvitge, IDIBELL (Institut D’investigació Biomèdica de Bellvitge), Universitat de Barcelona, Barcelona, Spain; 2 University Hospital, Universidade Federal do Rio de Janeiro, Rio de Janeiro, Brazil; 3 Hospital Universitari i Politecnic “La Fe”, Valencia, Spain; 4 Hospital Universitario “12 de Octubre”, Instituto de Investigación Hospital 12 de Octubre (i+12), Madrid, Spain; 5 Hospital Interzonal de Agudos “Dr. Rodolfo Rossi”, La Plata, Argentina; 6 Hospital “Dr. Guillermo Rawson”, San Juan, Argentina; University of Wisconsin Medical School, United States of America

## Abstract

**Background:**

Statins have immunomodulatory properties and hinder *Candida* growth. However, it is unknown whether they may improve prognosis in patients with candidemia. We sought to determine the effect of prior statin use on the clinical outcomes of patients suffering candidemia.

**Methods and Findings:**

Multicenter cohort study of hospitalized adults with candidemia between 2005 and 2011 in six hospitals in Spain, Brazil and Argentina. Of 326 candidemias, 44 (13.5%) occurred in statin users and 282 (86.5%) in statin non-users. The median value of APACHE II at candidemia diagnosis was similar between groups (18 vs. 16; p=.36). *Candida albicans* was the most commonly isolated species, followed by *C. parapsilosis*, *C. tropicalis*, *C. glabrata*, and *C. krusei*. There were no differences regarding appropriate empirical antifungal treatment. Statin users had a lower early (5 d) case-fatality rate than non-users (4.5 vs. 17%; p=.031). This effect was not observed with other cardiovascular drugs (aspirin, beta blockers and ACE inhibitors). Independent factor related to early case-fatality rate was APACHE II score (AOR, 1.08; 95% CI, 1.03–1.14; p=.002). An appropriate empirical antifungal therapy (AOR, 0.11; 95% CI, 0.04–0.26; p=<.001) and prior statin use were independently associated with lower early case-fatality (AOR, 0.17; 95% CI, 0.03–0.93; p=.041). Fourteen days (14d) and overall (30d) case-fatality rates were similar between groups (27% vs. 29%; p=0.77 and 40% vs. 44%; p=.66).

**Conclusions:**

The use of statins might have a beneficial effect on outcomes of patients with candidemia. This hypothesis deserves further evaluation in randomized trials.

## Introduction

Candidemia is a common cause of nosocomial bloodstream infections worldwide [1,2] and is associated with significant morbidity, prolonged hospital stay, high mortality and increased health care costs [3]. Importantly, some investigators report that overall mortality has not decreased over the past decades [1-4].

Hydroxymethylglutaryl-CoA reductase inhibitors, also known as statins, are increasingly used in clinical practice to treat dyslipidemia. Statin therapy has been shown to decrease cardiovascular events and mortality from coronary artery disease [5]. Interestingly, statins exhibit potent anti-inflammatory, anticoagulant, and anti-oxidative effects called “pleiotropic properties” [6]. Due to these properties, it has been suggested that these drugs may have beneficial effects during sepsis. Experimental and observational studies [7,8] have shown that statin therapy reduces inflammatory cytokines [9]. There is an increasing interest in determining whether statins improve prognosis of patients with severe infections. The results of certain observational studies suggest that statins may reduce mortality in patients suffering from sepsis [8,10,11], bacteremia [12], community-acquired bacterial pneumonia [7,13,14] and even influenza [15,16]. On the other hand, in vitro studies have found that statins have an intrinsic antifungal effect, hindering fungal growth [17]. 

However, information evaluating the effects of statin therapy on clinical outcomes of patients with candidemia is scarce. The purpose of this study is to assess whether prior statin use is associated with a decreased risk of mortality in a large multicenter cohort of adult patients with candidemia.

## Methods

### Setting, patients, and study design

We performed a retrospective multicenter study of all episodes of candidemia occurring in hospitalized adult patients between January 2005 and December 2011 at six tertiary teaching institutions in three different countries: three in Spain, two in Argentina and one in Brazil. Only the first episode of candidemia for each patient was analyzed. The following information was carefully collected from medical records: demographic characteristics, comorbidities, statin use and other concurrent cardiovascular medications (aspirin, beta blockers and angiotensin II-converting enzyme inhibitors), clinical features, sources of candidemia, causative species, antifungal therapy and outcomes. Episodes of candidemia occurring in statin users were compared with those occurring in statin non-users. To protect personal privacy, identifying information of each patient in the electronic database was encrypted. Informed consent was waived by the Clinical Research Ethics Committee because no intervention was involved and no patient identifying information was included.

### Definitions

Candidemia and catheter-related candidemia were defined on the basis of the guidelines of the Infectious Diseases Society of America [1]. Secondary candidemia was defined as a documented concurrent infection caused by the same *Candida* species at a site other than the catheter [18]. An episode of candidemia was considered to be nosocomially acquired, community-acquired or healthcare-associated as described elsewhere [19]. Statin use was considered to be present in those patients who were taking a statin (simvastatin, atorvastatin, lovastatin, pravastatin or rosuvastatin) within the 7 days prior to the candidemia episode. Seven days period was used due to pleitropic effects of statins may persist despite temporary cessation of administration. The use of other cardiovascular drugs like aspirin, beta-blockers and angiotensin II-converting enzyme (ACE) inhibitors was considered to be present in patients who were taking these drugs within the 30 days prior to the candidemia episode. The diagnosis of septic shock was based on a systolic blood pressure of less than 90 mm Hg and peripheral hypoperfusion with the need for vasopressors [20]. Neutropenia was considered when the granulocyte count was <500/ mm^3^. Empirical antifungal therapy was considered to be appropriate when the *Candida* isolates showed in vitro susceptibility to the antifungal drug administered. When antifungal susceptibility testing was not available, we considered fluconazole, amphotericin B or an equinocandin as appropriate empirical antifungal treatment for *Candida albicans*, *Candida tropicalis*, *Candida parapsilosis* and *Candida lusitaniae*. For *Candida glabrata* and *Candida krusei*, empirical antifungal treatment was considered to be appropriate when an equinocandin or amphotericin B was administered. The early, 14 days and overall case-fatality rates were defined as death from any cause within five, fourteen and 30 days after the onset of candidemia respectively.

### Microbiological studies

Two sets of two blood samples were drawn from patients with suspected bloodstream infection. Blood samples were processed by the BACTEC 9240 system (Becton Dickinson Microbiology Systems, Franklin Lakes, NJ, USA) with an incubation period of five days. If yeast cells were observed after microscopic examination of Gram stain, blood bottles were subcultured onto Sabouraud agar plates (BD BBL StrackerTM PlatesTM, Heidelberg, Germany) and chromogenic media (CAN2 ChromID™Candida Agar, BioMerieux, Paris, France). Yeast isolates were identified by conventional methods. In vitro antifungal activity was studied by a commercial microdilution method (YeastOne®Sensitre®, TREK Diagnostic Systems Ltd, England) or by E-test (BioMerieux SA, Paris, France), in accordance with the manufacturer’s instructions. Antifungal susceptibility of isolates was classified according to the Clinical and Laboratory Standards Institute M27-A3 document [21].

### Statistical analysis

The results were analyzed using SPSS version 15.0 (SPSS Inc., Chicago, IL, USA). To detect significant differences between groups, we used the Chi-square test or Fisher’s exact test for categorical variables and the Student t-test or Mann-Whitney test for continuous variables, as appropriate. We performed a multivariate logistic regression analysis of factors potentially associated with mortality included all variables that were significant in the univariate analysis. Due to the baseline imbalances between patients, a propensity score for receiving statin therapy was added to the model. The propensity score (PS), probability of receiving statins, was calculated using multivariate logistic regression model and included the following variables: age, Charlson index, place of acquisition (inpatient or outpatient, with the first as a reference), neutropenia and urinary catheter. The model showed a P value of 0,646 for the Hosmer-Lemeshow test and an area under curve of 0.73, showing good predictive ability. The relative risks were expressed as adjusted odds ratios (AOR) and 95% confidence intervals. Goodness-of-fit of the final model was assessed by the Hosmer-Lemeshow test. Statistical significance was established at α=0.05. All reported p-values are two-tailed. 

## Results

### Patient characteristics

Over the study period we documented 326 candidemias, 44 (13.5%) occurring in statin users and 282 (86.5%) in statin non-users. The epidemiological and clinical characteristics of the patients are outlined in Table 1. Comparing with statin non-users, statin users were older and more frequently had chronic renal disease, diabetes mellitus and chronic heart disease. The presence of a urinary catheter was also more frequent in this group. Conversely, statin users had less chronic liver disease and neutropenia. The median value of APACHE II at candidemia diagnosis was similar in the two groups. 

**Table 1:** Clinical characteristics of patients by statin group.

	**Statin users (n=44) No. (%)**	**Statin non-users (n=282) No. (%)**	***p*-value**
**Demographics**			
**Male sex**	24 (54.5)	165 (58.5)	.620
**Age, median (IQR) years**	64 (56-74)	57 (43-70)	**.006**
**Comorbid conditions**			
**Chronic renal disease**	18 (40.9)	63 (22.3)	**.008**
**Dialysis**	6 (13.6)	17 (6)	.067
**Diabetes mellitus**	22 (50)	67 (23.8)	**<.001**
**COPD**	10 (22.7)	57 (20.2)	.710
**Chronic heart disease**	25 (56.8)	61 (21.6)	<.001
**Cerebrovascular disease**	8 (18.2)	40 (14.2)	.505
**Liver disease**	2 (4.5)	52 (18.4)	**.021**
**Malignancy**	16 (36.4)	111 (39.4)	.692
**Stem cell transplantation**	2 (4.5)	20 (7.1)	.394
**Graft versus Host disease**	0 (0)	8 (2.8)	.273
**Solid organ transplantation**	6 (13.6)	26 (9.2)	.364
**HIV infection**	0 (0)	14 (5)	.131
**Risk factors**			
**ICU stay**	20 (45.5)	132 (46.8)	.802
**Mechanical ventilation**	18 (40.9)	103 (36.5)	.636
**Total parenteral nutrition**	13 (29.5)	88 (31.2)	.768
**Vasopressor therapy**	10 (22.7)	78 (27.7)	.430
**Previous surgery**	23 (52.3)	112 (39.7)	.120
**Catheter placement (>48h)**	41 (93.2)	248 (87.9)	.360
**Urinary catheter**	32 (72.7)	130 (46.1)	**.002**
**Neutropenia**	0 (0)	42 (14.9)	**.006**
**Chemotherapy**	4 (9.1)	58 (20.6)	.070
**Radiotherapy**	1 (2.3)	9 (3.2)	.735
**Corticosteroid therapy**	15 (34.1)	133 (47.2)	.122
**Prior antibiotic therapy**	41 (93.2)	256 (90.8)	.801
**Immunosuppressive therapy**	10 (22.7)	65 (23)	.953
**Antifungal prophylaxis**	2 (4.5)	35 (12.4)	.120
**Clinical characteristics (at candidemia diagnosis)**			
**Fever**	38 (86.4)	213 (75.5)	.169
**Hypotension**	26 (59.1)	125 (44.3)	.293
**Vasopressor therapy requirement**	16 (36.4)	83 (29.4)	.402
**Acute renal failure**	18 (40.9)	81 (28.7)	.127
**Confusion**	20 (45.5)	98 (34.8)	.217
**APACHE II, median (IQR)**	18 (11-23)	16 (11-23)	.365

### *Candida* species

Among the species, the most frequent was *Candida albicans* followed by *C. parapsilosis, C. tropicalis, C. glabrata*, and *C. krusei* without significant differences between statin users and statin non-users. The place of acquisition, the likely source of infection and the species isolated are detailed in Table 2.

**Table 2:** Sources of candidemia and *Candida* species according to statin groups.

	**Statin users (n=44) No. (%)**	**Statin non-users (n=282) No. (%)**	***p*-value**
Site of acquisition *			.018
**Nosocomially-acquired**	37 (84.1)	242 (85.8)	
**Community-acquired**	7 (15.9)	15 (5.3)	
**Healthcare-associated**	0 (0)	23 (8.2)	
**Source of infection**			.615
**Urinary tract**	8 (18.2)	29 (10.3)	
**Digestive tract and abdomen**	4 (9.1)	44 (15.6)	
**Catheter related infection**	9 (20.5)	64 (22.7)	
**Skin and soft tissue**	1 (2.3)	3 (1.1)	
**Surgical site infection**	1 (2.3)	3 (1.1)	
**Endocarditis**	0 (0)	3 (1.1)	
**Unknown**	21 (47.7)	134 (47.5)	
**Others**	0 (0)	2 (0.7)	
***Candida* species**			
***C. albicans***	21 (47.7)	121 (42.9)	.549
***C. parapsilosis***	13 (29.5)	53 (18.8)	.099
***C. glabrata***	3 (6.8)	32 (11.3)	.367
***C. tropicalis***	6 (13.6)	41 (14.5)	.874
***C. krusei***	1 (2.3)	11 (3.9)	.594
***C. lusitaniae***	0 (0)	2 (0.7)	.575
**Others**	0 (0)	22 (7.8)	.055

* The site of acquisition was not known in 2 patients.

### Treatment and outcomes

The treatment and clinical outcomes are detailed in Table 3. More than 70% of patients received an appropriate empirical antifungal treatment without significant differences between groups. The most frequently used drug was fluconazole, followed by anidulafungin. No significant differences were found in catheter removal between groups. No differences were observed in persistent candidemia, instability after 48 hours of treatment or septic metastases. The early case fatality rate was lower among statin users (n=2: 4.5% vs. n=48: 17%; p=.031). After adjusting for Propensity Score, the statins also decrease the probability of early case-fatality rate significantly (p = 0.014). This effect was not observed with any of the other cardiovascular drugs analyzed, including aspirin (odds ratio [OR], 1.5; 95% confidence interval [CI], 0.65–3.5; p=0.34), beta blockers (OR, 0.69; 95% CI, 0.23–2.0; p=0.49) or with ACE inhibitors (OR, 0.69; 95% CI, 0.3–1.5; p=0.36). Fourteen days (14d) and overall (30d) case-fatality rates were similar between groups (27% vs. 29%; p=0.77 and 40% vs. 44%; p=.66, respectively). The results were equivalent when neutropenic patients were excluded. 

**Table 3:** Treatments and clinical outcomes of patients by statin groups.

	**Statin users (n=44) No. (%)**	**Statin non-users (n=282) No. (%)**	***p*-value**
**Appropriate empirical antifungal treatment**	34 (77.3)	203 (71.9)	.464
**Antifungal drug selected**			.864
**Fluconazole**	21 (61.7)	117 (57.6)	
**Itraconazole**	0 (0)	1 (0.5)	
**Voriconazole**	1 (2.9)	7 (3.4)	
**Anidulafungin**	4 (11)	22 (10.8)	
**Caspofungin**	5 (15)	18 (8.8)	
**Micafungin**	0 (0)	10 (4.9)	
**Amphotericin B deoxycholate**	3 (8.8)	20 (9.8)	
**Liposomal Amphotericin B**	0 (0)	8 (3.9)	
**Catheter removal**	33 (75)	157 (55.7)	.138
**Outcomes**			
**ICU admission**	13 (29.5)	88 (31.2)	.734
**Mechanical ventilation**	12 (27.3)	85 (30.1)	.596
**Instability (at 48h)**	15 (34.1)	103 (36.5)	.840
**Persistent candidemia**	5 (11.4)	41 (14.5)	.645
**Septic metastases**	2 (4.5)	13 (4.6)	.529
**Early case-fatality rate (5d**)	2 (4.5)	48 (17)	**.031**
**Fourteen days case-fatality rate (14d**)	12 (27)	83 (29)	.77
**Overall case-fatality rate (30d)**	18 (40.1)	124 (44)	.663

### Independent factors associated with early case-fatality rate

Multivariate analysis adjusted for risk factors associated with mortality and including the propensity score is described in Table 4. The APACHE II score was an independent factors related to mortality (AOR, 1.08; 95% CI, 1.03-1.14; p=.002). An appropriate empirical antifungal therapy (AOR, 0.11; 95% CI, 0.04-0.26; p=<.001) and prior statin use were independently associated with lower early case-fatality (AOR, 0.17; 95% CI, 0.03–0.93; p=.041).

**Table 4:** Independent factors associated with early case-fatality rate: multivariate analysis.

**Characteristic**	**Adjusted odds ratio (AOR)**	**95% confidence interval**	***p*-value**
**Age**	0.98	0.95-1.005	.104
**Sex**	1.50	0.63-3.60	.360
**Charlson Comorbidity Index**	1.09	0.93-1.27	.307
**APACHE II score**	**1.08**	**1.03-1.14**	**.002**
**Aproppiate empirical antifungal therapy**	**0.11**	**0.04-0.26**	**<.001**
**PS**	**260**	**1.06-63525**	**.048**
**Statin therapy**	**0.17**	**0.03–0.93**	**.041**

## Discussion

In this multicenter study involving a large number of hospitalized adults with candidemia, we found that patients who had received statins had lower early case-fatality rate compared with those who were not receiving statins. Interestingly, the survival benefit observed persisted after adjustment for confounders by multivariate analysis.

To date, candidemia remains to be associated with significant morbidity and mortality. In the present study, the overall case-fatality rate exceeded 40%, similar to those reported in most series [3,22–26]. The early case-fatality rate was 4.5%. This figure is difficult to compare with others obtained in previous series because little information is available regarding frequency and associated factors. We found that APACHE II score was an independent factors related to mortality whereas an appropriate empirical antifungal therapy and prior statin use were independently associated with lower early case-fatality.

Statins were also associated with a lower overall (30d) case-fatality rate in ICU patients with candidemia in a previous single-center study conducted by Forrest et al [27]. That study had a small sample size (45 patients, including 15 statin users) and the exposure groups presented significant differences in APACHE II score. However, the overall survival benefit was not statistically significant when adjusted for APACHE II score. Another recent single-center study [28] did not show any benefit in the outcomes of patient with candidemia receiving statins. It was a small study (14 statin users) and did not evaluate the differences in the severity of the disease between groups. None of these studies analyzed the effect of statins in early mortality.

It might be speculated that the lower early case-fatality rates observed in statin users are due to the pleiotropic effects of statins. There is substantial evidence from basic science of the immunonodulatory role of statins in patients with sepsis, who present reductions in proinflammatory cytokines (TNF-α and IL-6) [9], induction of haem oxygenase, direct alteration of leucocyte-endothelial cell interaction and a reduction in the expression of MHC II [29]. Previous investigations have noted the role of statins in the maintenance of microvascular integrity with restoration of the normal endothelium functioning, and the inhibition of cell adhesion molecules [30–32]. Thus statins may have a critical role in the early course of candidemia. Moreover, statins have demonstrated a direct antifungal effect: the inhibition of HMG-CoA reductase affects the synthesis of ergosterol, which strongly inhibits the growth of *Candida* species. Statins can also cause deletions in the mitochondrial genome of yeasts, hampering fungal growth [17]. Furthermore, synergy between statins and fluconazole has been reported, although not at clinically achievable concentrations [33].

Our study did not shown significant differences in overall case fatality rate between statins users and non-statins users. It should be noted, however, that host factors are the most important factors related with late death in patients with infection [34–36]. Poor prognosis within the first 30-days of candidemia is a marker of the fragile status of patients with candidemia. Therefore, it seems reasonable that a potential immunomodulatory treatment have not effect in late deaths.

Some researchers have suggested that the beneficial effects of statins observed in infectious diseases may actually reflect a healthy user bias. If this was true, this “healthy user behaviour” would result in apparent benefit for all classes of cardiovascular drugs [7,13]. However, none of the concomitant cardiovascular drugs (aspirin, beta-blockers and ACE inhibitors) were independently associated with mortality in the present study. 

Our study has some limitations that should be noted. Firstly, it was retrospective and ha a small sample size of patients receiving statins. Secondly, most patients received empirical treatment with fluconazole. This practice may not necessarily reflect antifungal empirical choices at this time, after ESCMID recommendations for equinocandins use [37,38]. Thirdly, it did not specifically account for types of statins. Fourth, we also understand that the gut tolerance needed for statin administration could select a subgroup of patients in better conditions, even in the absence of differences in the APACHE II score between groups. Finally, the ideal timing for initiating statins with respect to the onset of sepsis is still unknown. Our patients were on chronic treatment with statins at onset of candidemia. The role of statins administered *de novo* in the context of a *Candida* sepsis should be analyzed in further studies. 

In conclusion, the results of this multicenter study with a large cohort of hospitalized patients showed that prior statins use may improve the early case fatality rate in patients with candidemia. However, overall mortality was not different between patients receiving statins and those without this drug. This early beneficial effect of statins deserves to be evaluated in randomized trials. 

## Ethics Approval

This retrospective observational study was conducted in accordance with the Declaration of Helsinki and was approved by the Institutional Review Board of the Comité Ético de Investigación Clínica del Hospital Universitari de Bellvitge (Clinical Research Ethics Committee, Hospital Universitari de Bellvitge). To protect personal privacy, identifying information of each patient in the electronic database was encrypted. Informed consent was waived by the Clinical Research Ethics Committee because no intervention was involved and no patient identifying information was included.
